# Assessing robustness of hazard ratio estimates to outcome misclassification in longitudinal panel studies with application to Alzheimer’s disease

**DOI:** 10.1371/journal.pone.0190107

**Published:** 2017-12-22

**Authors:** Le Wang, Rebecca A. Hubbard, Rod L. Walker, Edward B. Lee, Eric B. Larson, Paul K. Crane

**Affiliations:** 1 Department of Biostatistics, Epidemiology & Informatics, University of Pennsylvania, Philadelphia, PA, United States of America; 2 Kaiser Permanente Washington Health Research Institute, Seattle, WA, United States of America; 3 Department of Pathology & Laboratory Medicine, University of Pennsylvania, Philadelphia, PA, United States of America; 4 Department of Medicine, University of Washington, Seattle, WA, United States of America; Indiana University, UNITED STATES

## Abstract

Analyses of imperfectly assessed time to event outcomes give rise to biased hazard ratio estimates. This bias is a common challenge for studies of Alzheimer’s Disease (AD) because AD neuropathology can only be identified through brain autopsy and is therefore not available for most study participants. Clinical AD diagnosis, although more widely available, has imperfect sensitivity and specificity relative to AD neuropathology. In this study we present a sensitivity analysis approach using a bias-adjusted discrete proportional hazards model to quantify robustness of results to misclassification of a time to event outcome and apply this method to data from a longitudinal panel study of AD. Using data on 1,955 participants from the Adult Changes in Thought study we analyzed the association between average glucose level and AD neuropathology and conducted sensitivity analyses to explore how estimated hazard ratios varied according to AD classification accuracy. Unadjusted hazard ratios were closer to the null than estimates obtained under most scenarios for misclassification investigated. Confidence interval estimates from the unadjusted model were substantially underestimated compared to adjusted estimates. This study demonstrates the importance of exploring outcome misclassification in time to event analyses and provides an approach that can be undertaken without requiring validation data.

## Introduction

Estimates of the relationship between time to event outcomes and exposures are biased in the presence of imperfect ascertainment of the outcome of interest. When misclassification in the outcome is small and independent of predictor variables, the effect of misclassification on the measure of association is correspondingly small and towards the null. However, when misclassification is differential, estimates of association that do not account for misclassification may be attenuated towards or away from the null [1, 2]. Past work has demonstrated that by incorporating the sensitivity and specificity of the imperfect outcome into the analysis, the true association can be recovered and unbiased estimates of the association between disease and risk factors can be obtained [1–4]. Bias-corrected estimators have been developed using discrete proportional hazards models [5, 6], a particularly appealing approach for studies under longitudinal panel observation because it addresses the interval censored nature of the outcome [7] and the structure of the analytic model mirrors the structure of outcome assessment which occurs at equally distributed discrete time-points. Discrete time models have been developed to address interval censored outcomes in a variety of contexts (e.g., [8–10]).

Studies that investigate risk factors for development of Alzheimer’s disease (AD) neuropathology using clinical diagnoses of AD provide an example of a context in which outcome misclassification is common. Accounting for outcome misclassification in the context of AD neuropathology is particularly challenging due to the complex relationship between the clinically observable phenotype and the underlying pathophysiology. While many research studies use the National Institute of Neurological Disorders and Stroke-Alzheimer Disease and Related Disorders (NINCDS-ADRDA) criteria [11] for clinical diagnosis of AD, more recent diagnostic criteria have emphasized the distinction between the clinical disease and the underlying AD neuropathology [12–14]. Patients with biomarkers indicative of AD neuropathology may be asymptomatic while those with cognitive impairment or dementia may have one of numerous other conditions that manifest in memory deficits. Thus even sensitivity and specificity of diagnostic criteria based on symptoms and biomarkers are imperfect with respect to the underlying pathology.

The objective of this study was to demonstrate how existing statistical approaches accounting for outcome misclassification in the context of a time to event analysis can be used to evaluate robustness of study results to misclassification, even in the absence of a validation sub-sample. To exemplify this sensitivity analysis approach, we used data from the Adult Changes in Thought (ACT) study, a longitudinal panel study of older adults with serial assessment of cognitive functioning and AD risk factors. Data from this study previously were used to identify a statistically significant positive association between average glucose levels and dementia risk in individuals with and without diabetes [15]. Using this same cohort, we investigated the association between average glucose levels and AD neuropathology, demonstrating the effect of outcome misclassification resulting from the use of clinical diagnosis data to make inference about risk factors for underlying neuropathologic changes.

## Materials and methods

### Overview of time to event outcomes under panel observation

A panel study is a longitudinal study featuring repeated assessment of a cohort of subjects at a pre-defined sequence of time points often referred to as study waves. This common epidemiological study design has been used frequently to investigate risk factors associated with AD [16–19]. In this study design, a cohort of participants is followed longitudinally with periodic assessment of outcomes at discrete time-points. For instance, in the context of AD, participants may receive annual or biennial study visits at which cognitive testing is carried out to determine AD status. Such studies give rise to survival data of the form {*t*_*i*_, *d*_*i*_} where *t*_*i*_ is the earlier of the time of the event of interest or a censoring time if the study ends or the participant is lost to follow-up and *d*_*i*_ is a binary indicator taking the value 1 if the participant experienced an event and 0 otherwise. Survival data of this form are often analyzed using the Cox proportional hazards model. This approach allows for right censoring of time to event data. However, in its standard form it does not address interval censoring arising because AD status is only available at discrete study follow-up visits, although the true onset time of clinical AD lies somewhere in the interval between visits. Applying the standard Cox proportional hazards model without accounting for interval censoring can lead to erroneous inference [20]. Additional modification of the standard approach is also needed if the assessment for the outcome of interest is imperfect. In the case of AD, clinical AD diagnosis corresponds imperfectly with the presence of AD neuropathology. Thus hazard ratio estimates based on clinical AD diagnosis will be biased for hazard ratios describing the association between exposures and underlying AD neuropathology.

Below we discuss the discrete proportional hazards model as one approach to address the interval censored nature of longitudinal cohort data under panel observation [7–9] and an extension of this approach developed by Meier et al. [6] to further accommodate outcome misclassification. We then illustrate how this approach can be used to explore sensitivity of results to outcome misclassification when validation data may or may not be available for a subset of participants.

### Discrete proportional hazards model

The discrete proportional hazards model [21] is appropriate for outcomes that are assessed at periodic study visits separated by equal length time intervals such as those encountered in studies under panel observation and has been widely used to analyze interval-censored data in aging and dementia studies [22–25]. In this model, the baseline hazards are given by **λ_0_** = (λ_01_, λ_02_, …, λ_0*T*_)^*T*^ at time 1 to *T*. The hazard for the *i*th subject at time *j* with covariates ***X*_*i*_** is $1-{\left(1-\lambda_{0j}\right)}^{e^{X_{i}^{\prime }\beta }}$, and we can write the likelihood for the *i*th subject as

$$\begin{array}{c} \begin{array}{c} f\left(t_{i},d_{i};X_{i},\beta ,\lambda_{0}\right)=\left\{\prod_{j=1}^{t_{i}-1}{\left(1-\lambda_{0j}\right)}^{e^{\left(X_{i}^{\prime }\beta \right)}}\right\}\times {\left\{1-{\left(1-\lambda_{0t_{i}}\right)}^{e^{\left(X_{i}^{\prime }\beta \right)}}\right\}}^{d_{i}}\times {\left\{{\left(1-\lambda_{0t_{i}}\right)}^{e^{\left(X_{i}^{\prime }\beta \right)}}\right\}}^{\left(1-d_{i}\right)}. \end{array} \end{array}$$ (1)

In this likelihood, the first term denotes the probability that no event occurs at study visits 1 to *t*_*i*_ − 1, the second term represents the likelihood contribution if an event occurred at the final study visit, and the final term denotes the likelihood contribution for censored participants where no event occurred at the final study visit. We can estimate regression parameters *β* and baseline hazards **λ_0_** using standard software for generalized linear models for binomial family data with complementary log-log link [26]. When **λ_0_** is small, *e*^*β*^ approximates the familiar hazard ratio from the Cox proportional hazards model.

### Adjusted discrete proportional hazards model

The adjusted discrete proportional hazards model extends the above approach to account for outcome misclassification by incorporating sensitivity (*θ*) and specificity (*ϕ*) of diagnostic tests [6]. Let *θ* and *ϕ* denote the sensitivity and specificity, respectively, of an imperfect outcome, such as a clinical AD diagnosis relative to the underlying neuropathology, which we assume is performed repeatedly over the course of longitudinal follow-up. Assume *t*_*i*_ is the true event time and $t_{i}^{o}$ is the observed event time. We further assume that once a subject is observed to have experienced an event, follow-up ends. The true event status, *d*_*i*_, is not observed. Instead $d_{i}^{o}$, an imperfect event status indicator, is available and takes the value 1 if the imperfect outcome occurs before the end of study follow-up and 0 otherwise.

Below we illustrate a sample observation pattern for a participant in a study of AD who developed AD neuropathology at time *t*_*i*_ and a clinical diagnosis of AD at time $t_{i}^{o}$.

$$\underset{t_{i}-1\;\text{true}\;\text{negatives}\;}{\underset{︸}{1,2,\cdots ,t_{i}-1,}}\underset{t_{i}^{o}-t_{i}\;\text{false}\;\text{negatives}}{\underset{︸}{\overset{{}_{\text{onset}}^{\;\text{AD}}}{\overset{︷}{t_{i}}},\cdots ,t_{i}^{o}-1,}}\overset{{}_{\text{diagnosis}}^{\;\text{AD}}}{\overset{︷}{t_{i}^{o}}}$$

We can express the probability of the observed event time and status conditional on the true underlying event time, *t*_*i*_ by noting that this pattern of observations corresponds to *t*_*i*_ − 1 true negative observations followed by $t_{i}^{o}-t_{i}$ false negatives and a single true positive observation at time $t_{i}^{o}$. In terms of the sensitivity and specificity of clinical diagnosis relative to the underlying pathology, the probability of this pattern of observations can be expressed as $\phi^{t_{i}-1}{\left(1-\theta \right)}^{t_{i}^{o}-t_{i}}\theta$.

We denote the probability of the observed imperfect event time and event status indicator conditional on the underlying true event time using Γ_*i*_ and Δ_*i*_, where

$$\begin{array}{ccc} f(t_{i}^{o},d_{i}^{o}|t_{i}=t_{i}^{o},d_{i}=0,\theta ,\phi ) & = & \phi^{t_{i}^{o}-1}\phi^{1-d_{i}^{o}}{\left(1-\phi \right)}^{d_{i}^{o}}≐\Gamma_{i}, \end{array}$$ (2)

$$\begin{array}{ccc} f(t_{i}^{o},d_{i}^{o}|t_{i}\leq t_{i}^{o},d_{i}=1,\theta ,\phi ) & = & \phi^{t_{i}-1}{\left(1-\theta \right)}^{t_{i}^{o}-t_{i}}{\left(1-\theta \right)}^{1-d_{i}^{o}}\theta^{d_{i}^{o}}≐\Delta_{it_{i}}. \end{array}$$ (3)

As shown by Meier et al. [6], we can express the likelihood for subject *i* accounting for misclassification by averaging over the distribution of unobserved true event times and event status,

$$\begin{array}{cc} f(t_{i}^{o},d_{i}^{o};X_{i},\beta ,\lambda_{0},\theta ,\phi ) & =[\prod_{j=1}^{t_{i}^{o}}{\left(1-\lambda_{0j}\right)}^{e^{\left(X_{i}^{\prime }\beta \right)}}]\Gamma_{i}+[1-{\left(1-\lambda_{01}\right)}^{e^{\left(X_{i}^{\prime }\beta \right)}}]\Delta_{i1}\\ & +\sum_{k=2}^{t_{i}^{o}}[\{\prod_{j=1}^{k-1}{\left(1-\lambda_{0j}\right)}^{e^{\left(X_{i}^{\prime }\beta \right)}}\}\times \{1-{\left(1-\lambda_{0k}\right)}^{e^{\left(X_{i}^{\prime }\beta \right)}}\}\Delta_{ik}]. \end{array}$$ (4)

We obtain outcome misclassification-adjusted estimates of *β* by numerically maximizing the likelihood function over {*β*, **λ_0_**}.

### Extensions to the case of a single gold-standard assessment

The misclassification adjusted discrete proportional hazards model assumes that study visit-level sensitivity and specificity, *θ* and *ϕ*, are available. However, in many studies under panel observation only a single assessment of the gold-standard outcome is possible. In this case, validation data on agreement between the proxy and gold standard outcome are only available aggregated across study follow-up, not at the level of the individual follow-up visit. For instance, in studies of AD, it is possible to conduct autopsies of deceased participants and ascertain agreement between AD neuropathology and a clinical diagnosis of AD prior to death. However, it is not possible to estimate the probability of a clinical AD diagnosis at each individual study visit conditional on the presence of underlying AD neuropathology at that study visit. Such information cannot be obtained because it is only possible to make a single determination of presence or absence of AD neuropathology on the basis of autopsy. Since the precise timing of the development of AD neuropathology is unknown, it is also unknown whether any individual study visit resulted in a correct or incorrect diagnosis. This challenge exists for studies of any disease where outcome validation can only be performed once at the end of study follow-up.

Using information on concordance between a single validated outcome at the end of study follow-up and the imperfect assessment of the event of interest during study follow-up, $d_{i}^{o}$, we can obtain estimates of *θ* and *ϕ* by assuming constant sensitivity and specificity of the imperfect assessment across follow-up. Specifically, for an individual who truly experienced the event of interest, we can write the likelihood for *θ* and *ϕ*, the visit-level sensitivity and specificity as

$$\begin{array}{c} L_{i}\left(\phi ,\theta \right)=\frac{1}{t_{i}^{o}}\sum_{j=1}^{t_{i}^{o}}\phi^{j-1}{\left(1-\theta \right)}^{t_{i}^{o}-j}\theta^{d_{i}^{o}}{\left(1-\theta \right)}^{1-d_{i}^{o}}. \end{array}$$ (5)

Note that this expression makes use of the simplifying assumption that true event occurrence was equally likely at any study visit prior to death. While this assumption is unlikely to hold in general, in the absence of data to support proposed alternative functional forms for the relationship between time and classification accuracy, it provides a convenient baseline model from which to begin exploring misclassification. In cases where data or proposed biologic mechanisms support alternative relationships, the above model can be modified to accommodate alternative specifications by replacing *ϕ* and *θ* with functions of time.

For an individual who truly did not experience the event of interest, the likelihood takes the form $L_{i}\left(\phi ,\theta \right)=\phi^{t_{i}^{o}-1}{\left(1-\phi \right)}^{d_{i}^{o}}\phi^{1-d_{i}^{o}}=\phi^{t_{i}^{o}-d_{i}^{o}}{\left(1-\phi \right)}^{d_{i}^{o}}$. We can thus express the likelihood for the complete validation sub-sample as

$$\begin{array}{c} L\left(\phi ,\theta \right)={\prod_{i=1}^{n}\left[\frac{1}{t_{i}^{o}}\sum_{j=1}^{t_{i}^{o}}\phi^{j}{\left(1-\theta \right)}^{t_{i}^{o}-j}\theta^{d_{i}^{o}}{\left(1-\theta \right)}^{1-d_{i}^{o}}\right]}^{d_{i}}{\left[\phi^{t_{i}^{o}-d_{i}^{o}}{\left(1-\phi \right)}^{d_{i}^{o}}\right]}^{1-d_{i}}. \end{array}$$ (6)

This likelihood can be maximized to obtain estimates for *ϕ* and *θ*.

### Sensitivity to outcome misclassification

Estimates for the hazard ratio based on numerical maximization of eq 4 are conditional on assumed values for assessment-level sensitivity and specificity. If sensitivity and specificity are known then these can be incorporated into estimation and adjusted hazard ratio estimates can be obtained. In many cases no validation data or only a small validation sub-sample may be available in which case it is preferable to investigate hazard ratio estimates under a range of values for sensitivity and specificity. By specifying a plausible range for sensitivity and a plausible range for specificity we can construct a grid of sensitivity and specificity values and obtain hazard ratio estimates at each point in the grid. By examining variation in hazard ratio estimates across values for sensitivity and specificity we can explore robustness of estimates to imperfect outcome ascertainment. Additionally, confidence interval widths from the misclassification adjusted model can be compared to unadjusted confidence intervals to quantify the degree to which precision has been overestimated by ignoring outcome misclassification. The width of confidence intervals from the adjusted models is expected to be slightly smaller than the nominal level due to uncertainty in the estimated sensitivity and specificity. Comparison of adjusted and unadjusted confidence interval widths thus represents a lower bound for the overestimation of precision of the unadjusted approach.

### ACT study

The Adult Changes in Thought (ACT) study is an ongoing, longitudinal study of incident dementia. Participants were dementia-free, at least 65 years old at the time of enrollment, and randomly selected from Kaiser Permanente Washington (formerly Group Health), an integrated health care system in Washington-state. Study procedures have been previously described [27]. The study enrolled 2,581 participants between 1994 and 1996 [27], and an additional 811 participants were enrolled from 2000 through 2002 [15]. The ACT study followed the Helsinki declaration and was reviewed and approved by the Kaiser Permanente Washington and University of Washington institutional review boards. Written informed consent was obtained from all participants. Our analysis was based on a de-identified subset consisting of 1,955 participants who met the same inclusion criteria as a prior study of glucose and dementia [15] and were censored at age 89 years in order to satisfy HIPAA requirements for data de-identification.

Serial cognitive testing was performed every two years for the purpose of clinical diagnosis of dementia. The Cognitive Abilities Screening Instrument was used, where the score ranges from 0 to 100 and a higher score indicates better cognitive function [28]. Participants who had scores of 85 or below received further clinical and psychometric tests and the results of all evaluation, laboratory results, and image records were combined to reach a clinical diagnosis of possible or probable AD based on research criteria [11].

About one quarter of the cohort who died underwent brain autopsy and extensive pathological evaluation. We defined a binary indicator of the presence of AD neuropathology using a modified version of the National Institute on Aging-Reagan Institute criteria [12]. An individual was defined as having AD neuropathology if they had autopsy findings of Braak Stages V-VI and CERAD neuritic plaque frequency of “moderate” and “frequent”.

A variety of demographic and other exposure measures are available in the ACT data. To illustrate the use of the adjusted discrete proportional hazards model, we investigated the association between glucose levels and development of AD neuropathology. Time-varying glucose levels were determined by combining clinical measurements of glucose levels, glycated hemoglobin levels, and hemoglobin A1c levels, as previously described [15]. Average glucose levels were computed for each participant at study baseline and in 5-year rolling windows. Potential confounders of the relationship between incident AD neuropathology and glucose levels were captured using the ACT study and Kaiser Permanente Washington data sources. Blood pressures were averaged over two measurements separated by a five-minute rest period. Kaiser Permanente Washington pharmacy data were used to assess treatment for hypertension and diabetes.

### Statistical analysis

We first fit a discrete proportional hazards model to investigate the association between clinical diagnosis of possible or probable AD and quartiles of average glucose in participants with and without diabetes, including covariates ACT cohort, age at baseline, sex, treated hypertension status, and education level (at least a college education versus otherwise). We included an interaction term between average glucose and diabetes status to facilitate separate estimation of glucose hazard ratios for individuals with and without diabetes. In this model, we directly used clinical AD diagnosis of possible or probable AD to define the outcome of interest and did not account for misclassification of this outcome with respect to AD neuropathology, the target of inference. We then applied the adjusted discrete proportional hazards model to account for outcome misclassification by incorporating the sensitivity (*θ*) and specificity (*ϕ*) of clinical AD diagnosis relative to presence of AD neuropathology at autopsy. We estimated sensitivity and specificity of clinical diagnosis at each study visit using the likelihood-based procedure described above. We calculated Γ_*i*_ for each subject and Δ_*ij*_ for each subject at each time point. In order to investigate the impact of outcome misclassification, quantified by sensitivity (*θ*) and specificity (*ϕ*), on the estimates of the association between development of AD neuropathology and the average glucose level using the adjusted approach, we conducted sensitivity analyses varying sensitivity and specificity across plausible ranges suggested by analysis of data from the autopsy cohort and present the estimated hazard ratios across these ranges. We considered values for *θ* ranging from 0.3 to 0.5 with *ϕ* fixed at 0.97, and varied *ϕ* from 0.97 to 0.99 with *θ* fixed at 0.35. These ranges were selected based on results of analyses of the autopsy sub-sample.

## Results

Clinical and demographic characteristics of the ACT study sample at last clinical assessment, overall and stratified by the availability of autopsy data, are presented in Table 1. Among 1,955 participants, 148 were autopsied. The median glucose level (interquartile range, IQR) was 164.3 mg/dl (147.5-186.0 mg/dl) among participants with diabetes and 101.7 mg/dl (96.7-108.4 mg/dl) among participants without diabetes. The median age was 75 years and 59% of the cohort was female. The study sample was pre-dominantly white (89.9%), and 60% of participants had at least a college education. Autopsied participants tended to be slightly older than non-autopsied participants at baseline. A greater proportion of autopsied participants were white and developed clinical dementia or a clinical diagnosis of possible or probable AD.

**Table 1:** Demographic and clinical characteristics of ACT study participants at last clinical assessment stratified by availability of autopsy data.

	Overall (N = 1,955)	Autopsied (N = 148)	Non-autopsied (N = 1,807)
Original study cohort, N (%) ^a^			
No	524 (26.8)	31 (20.9)	493 (27.3)
Yes	1,431 (73.2)	117 (79.1)	1,314 (72.7)
Age at baseline, median (IQR) ^b^	75 (71, 80)	76 (73, 79)	74 (70, 80)
Female, N (%)			
No	810 (41.4)	65 (43.9)	745 (41.2)
Yes	1,145 (58.6)	83 (56.1)	1,062 (58.8)
Non-white, N (%)			
No	1,757 (89.9)	143 (96.6)	1,614 (89.3)
Yes	198 (10.1)	5 (3.4)	193 (10.7)
College education, N (%)			
No	780 (39.9)	58 (39.2)	722 (40.0)
Yes	1,175 (60.1)	90 (60.8)	1,085 (60.0)
*APOEϵ*4+, N (%)			
No	1,288 (74.8)	95 (69.9)	1,193 (75.2)
Yes	434 (25.2)	41 (30.1)	393 (24.8)
*Missing*	233	12	221
Average systolic BP, median (IQR)	137 (123, 151)	130 (118, 143)	138 (123, 151)
Average diastolic BP, median (IQR)	70 (63, 79)	70 (62, 75)	70 (63, 79)
Treated hypertension, N (%)			
No	307 (15.7)	23 (15.5)	284 (15.7)
Yes	1,648 (84.3)	125 (84.5)	1,523 (84.3)
Glucose, median (IQR)			
Diabetes	164.3 (147.5, 186)	159.2 (141.7, 190.1)	164.5 (147.9, 185.2)
No Diabetes	101.7 (96.7, 108.4)	102.4 (97.1, 110.0)	101.6 (96.7, 108.0)
Clinical dementia, N (%)			
No	1,557 (79.6)	103 (69.6)	1,454 (80.5)
Yes	398 (20.4)	45 (30.4)	353 (19.5)
Clinical possible/probable AD, N (%)			
No	1,657 (84.8)	116 (78.4)	1,541 (85.3)
Yes	298 (15.2)	32 (21.6)	266 (14.7)

Abbreviations: AD, Alzheimer’s Disease; APOE*ϵ*4+, presence of at least one *ϵ*4 allele in the apolipoprotein E genotype; BP, blood pressure; IQR, interquartile ranges.

^a^ Counts and percentages are presented for categorical variables. Percentages are computed among all non-missing values.

^b^ Medians and interquartile ranges (IQR) are presented for continuous variables.

Among subjects with available autopsy data, 20 were classified as meeting neuropathological criteria for AD and 128 did not meet criteria for AD. Sensitivity and specificity of a clinical AD diagnosis relative to AD neuropathology were modest. Among those with AD neuropathology, 55% (95% confidence interval [CI] 33.6, 74.7) had a clinical diagnosis of possible or probable AD. Among autopsied participants without AD neuropathology, 83.6% (95% CI 76.1, 89.1) did not have a clinical diagnosis of possible or probable AD. Based on these sensitivity and specificity values which aggregate information across all study follow-up visits, we computed the estimated assessment-level sensitivity (*θ*) and specificity (*ϕ*) to be 36.2% and 94.5% respectively.

Based on an unadjusted discrete proportional hazards analysis, the hazard of a clinical AD diagnosis did not differ across quartiles of average glucose level in the prior five years for participants with or without diabetes (Table 2). For participants without diabetes, hazard ratios for each glucose quartile relative to the lowest quartile of glucose exposure were all greater than one, indicating non-statistically significantly increased hazard of AD among participants with higher glucose levels. For participants with diabetes, hazard ratios were less than one for the second and third quartiles of glucose exposure relative to the lowest while the hazard ratio for the fourth quartile was greater than one. These differences were also not statistically significant. We next estimated hazard ratios using the adjusted discrete proportional hazards model with values for *θ* and *ϕ* based on results from our autopsy sub-sample. Because the likelihood became extremely flat as specificity decreased it was not possible to obtain maximum likelihood estimates for values of *ϕ* < 0.97. We therefore report estimates at specificity of 0.97 as this was closest to the point estimate of 0.945 obtained based on the autopsy cohort at which the numerical maximization algorithm converged. Compared to unadjusted estimates, adjusted hazard ratio estimates assuming *θ* = 0.35 and *ϕ* = 0.97 were generally larger in magnitude, although far less precisely estimated (Table 2).

**Table 2:** Hazard ratios and 95% confidence intervals for association between glucose level and AD diagnosis based on discrete proportional hazards model.

	Unadjusted HR ^a^ (95% CI)	Adjusted HR ^b^ (95% CI)	Difference in HR	Relative CI width ^c^
No diabetes				
Q2 (95.9-100.9)	1.04 (0.72 1.50)	1.82 (0.80 4.17)	0.78	4.3
Q3 (100.9-107.8)	1.21 (0.85 1.73)	1.84 (0.72 4.72)	0.63	4.5
Q4 (>107.8)	1.28 (0.90 1.82)	2.13 (0.82 5.55)	0.85	5.1
Diabetes				
Q2 (149.5-167.0)	0.86 (0.40 1.86)	0.30 (0.02 15.48)	-0.56	3.7
Q3 (167-187.7)	0.59 (0.25 1.41)	0.68 (0.08 15.59)	-0.09	4.8
Q4 (>186.7)	1.19 (0.54 2.62)	2.41 (0.42 13.76)	-1.22	6.4

Abbreviations: AD, Alzheimer’s Disease; CI, confidence intervals; HR, hazard ratios; Q2, the second quartle; Q3, the third quartile; Q4, the fourth quartile

^a^ Unadjusted estimates do not account for outcome misclassification. The model additionally include covariates ACT study cohort, age at baseline, sex, college education, and treated hypertension.

^b^ Adjusted estimates use assumed value of *θ* = 0.35 and *ϕ* = 0.97. The model additionally include covariates ACT study cohort, age at baseline, sex, college education, and treated hypertension.

^c^ Relative CI width is the ratio of the adjusted 95% CI width to the unadjusted width.

**Fig 1:**
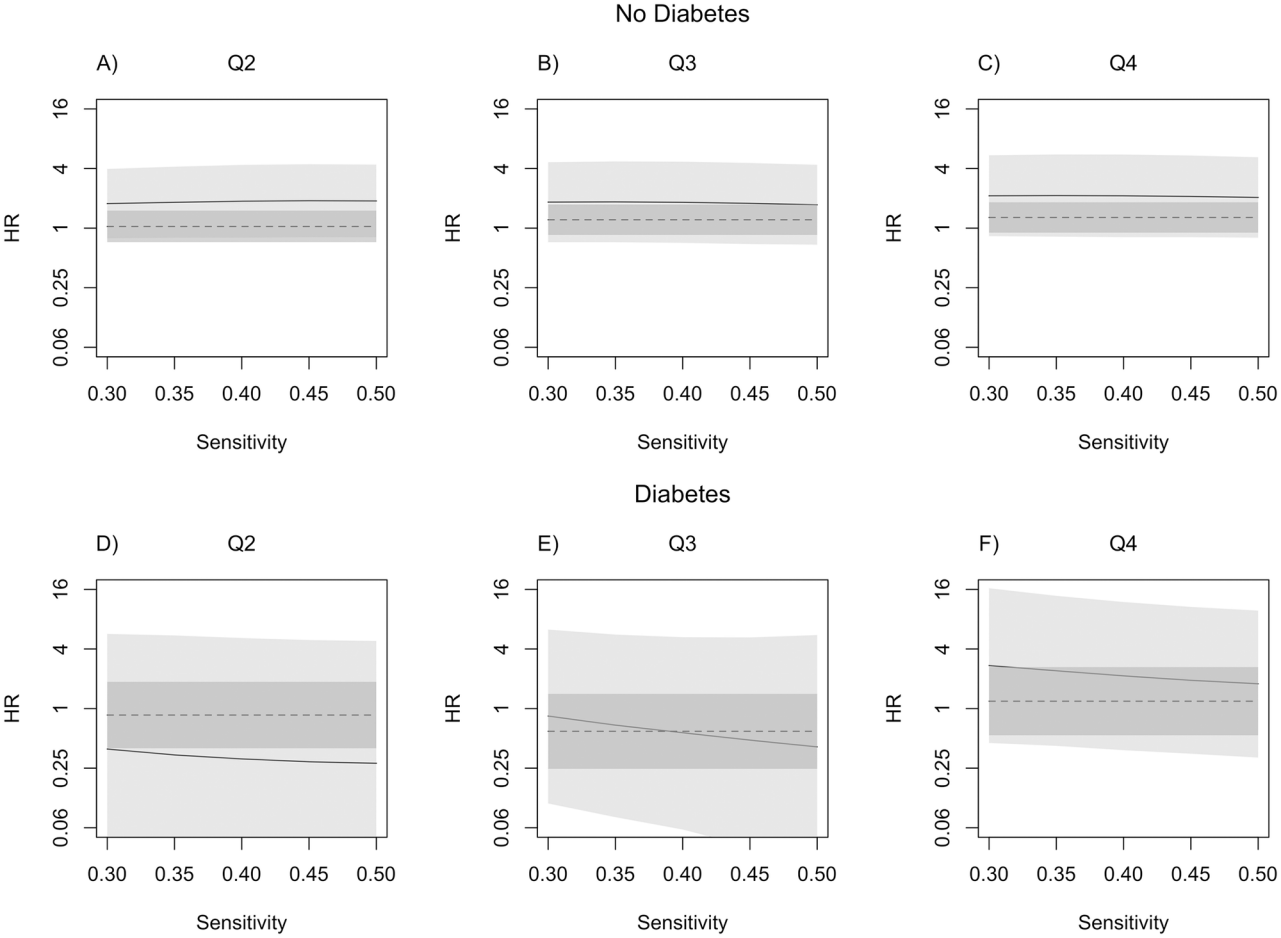
Adjusted hazard ratios (HR, solid line) and 95% confidence intervals (CI, light gray) for glucose quartiles 2-4 (Q2-Q4) relative to quartile 1 for varying sensitivities of clinical AD diagnosis (*θ*) with specificity (*ϕ*) fixed at 0.97. Dashed line represents unadjusted hazard ratio estimate and dark grey band represents unadjusted 95% CI.

**Fig 2:**
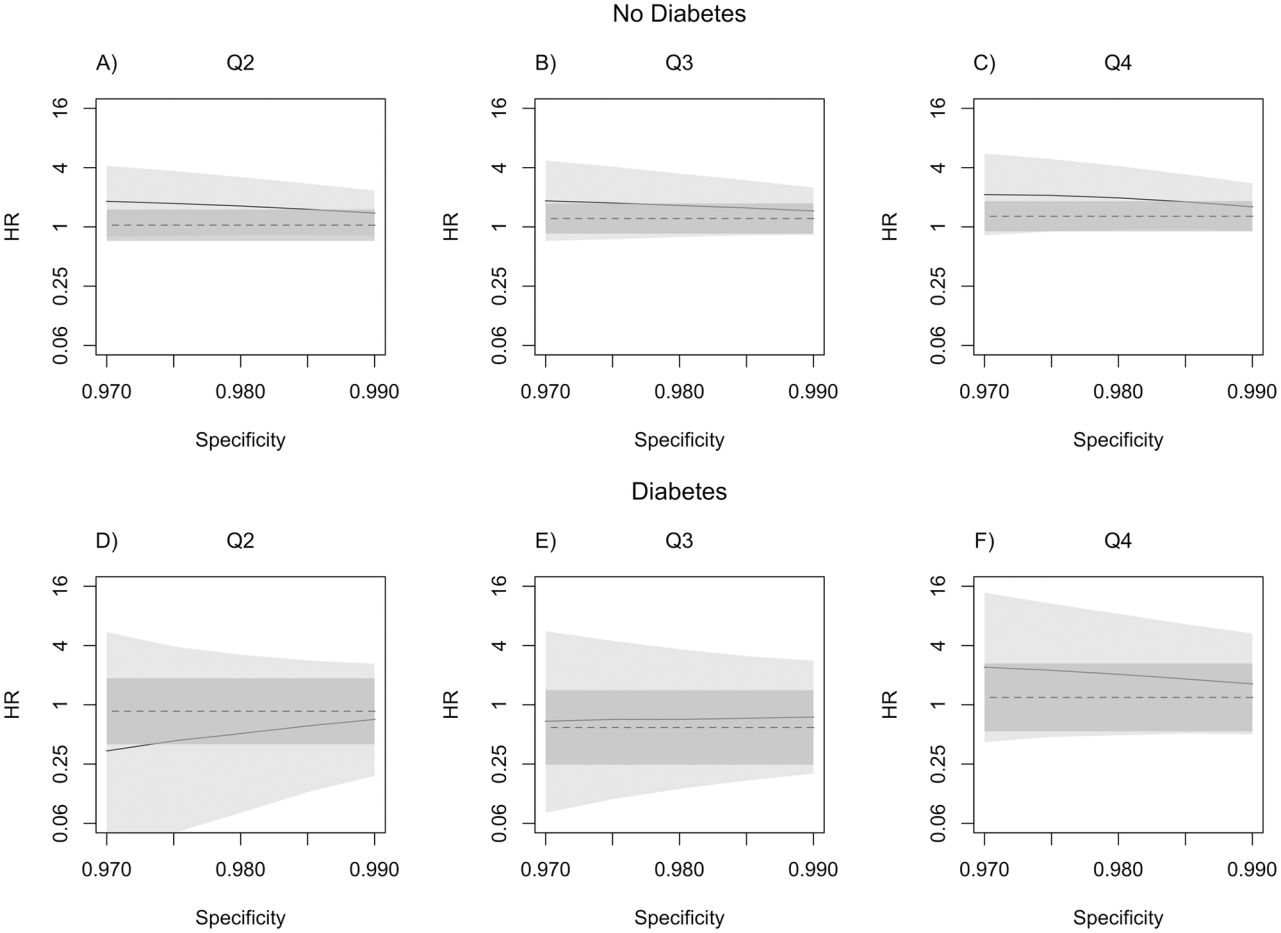
Adjusted hazard ratios (HR, solid line) and 95% confidence intervals (CI, light gray) for glucose quartiles 2-4 (Q2-Q4) relative to quartile 1 for varying specificities of clinical AD diagnosis (*ϕ*) with sensitivity (*θ*) fixed at 0.35. Dashed line represents unadjusted hazard ratio estimate and dark grey band represents unadjusted 95% CI.

Fig 1 presents the adjusted hazard ratio estimates for the second to fourth quartiles of the average glucose level in the prior 5 years relative to the first quartile of average glucose level, when varying sensitivity from 0.3 to 0.5 with specificity fixed at 0.97. Relative to adjusted estimates, the unadjusted hazard ratio estimates were attenuated towards the null across the range of values for sensitivity investigated for all parameters except the hazard ratio for quartile 3 for individuals with diabetes. Fig 2 shows the adjusted hazard ratio estimates for varying specificity from 0.97 to 0.99 with sensitivity fixed at 0.35. All unadjusted hazard ratio estimates were attenuated towards the null relative to the adjusted estimates except the hazard ratio for quartile 3 for individuals with diabetes. The hazard ratio estimates were more robust to sensitivity changes than specificity changes. Confidence interval widths increased dramatically as sensitivity and specificity decreased. Compared to unadjusted estimates, adjusted hazard ratios estimated at sensitivity of 0.35 and specificity of 0.97 had confidence interval widths that were more than four times wider (Table 2).

## Discussion

Outcome misclassification is common in epidemiologic studies, particularly those where the gold-standard diagnosis is difficult or expensive to obtain. AD neuropathologic change represents a particularly challenging outcome to assess since definitive diagnosis is only possible following death. We have demonstrated a method to account for outcome misclassification in time-to-event studies that has particular relevance for longitudinal studies under panel observation in which an imperfect outcome is ascertained at pre-determined periodic clinic visits. If participants are not assessed at common time points, for instance due to non-compliance with the study protocol, alternative methods for interval censored data are required (e.g., Zhang et al., 2010 [29]). In addition to extending the methods of Meier et al. [6] to the context where operating characteristics of the imperfect assessment are not available for individual assessments but only at the end of all study follow-up, we have also demonstrated how this approach can be used to conduct sensitivity analyses to evaluate robustness of study results to possible outcome misclassification. This approach addresses bias in studies with imperfect survival outcomes and also facilitates exploration of the effect of misclassification on precision of hazard ratio estimates.

Many studies of AD use a clinical diagnosis of possible or probable AD as the outcome of interest. This clinically assessed diagnosis is known to have imperfect sensitivity and specificity relative to the underlying AD neuropathology, and ignoring these imperfect operating characteristics introduces bias into estimated associations between risk factors and AD neuropathology. It is important to note that if the target of inference is the effect of risk factors on clinical diagnosis of AD or dementia then standard methods provide an unbiased estimate of these relationships. However, because dementia is a complex clinical syndrome arising through multiple etiologic pathways, estimating relationships with underlying neuropathologic changes may be more useful for elucidating biologic mechanisms. When associations with underlying neuropathology are of interest, using clinical data provides access to a larger study sample than analyses restricted to autopsied individuals but imperfect outcome assessment must be addressed to avoid bias.

Data from the ACT study have been used previously to investigate the association between glycemia and all-cause dementia. A prior study found that higher glucose levels were associated with increased hazard of dementia for individuals with and without diabetes [15]. A second study using only data on individuals with available autopsy data and no diabetes diagnosis found no association between glucose levels and extent of either neurofibrillary tangles or neuritic plaques [30]. Our study which combines clinical and autopsy data by treating clinical AD diagnosis as an imperfect proxy for underlying AD neuropathology similarly identified no statistically significant association between glucose level and AD neuropathology. Our analysis extends the prior work by allowing us to investigate an outcome ascertained at autopsy while incorporating information from both autopsied and non-autopsied participants. Sensitivity analyses making use of the adjusted discrete proportional hazards model indicated that both positive and negative effects were likely attenuated towards the null due to outcome misclassification. However, these results also indicate that the unadjusted analysis substantially overestimates the precision of the hazard ratio estimates by ignoring uncertainty in the outcome. Estimates based on assumed values for sensitivity and specificity derived from an autopsy sub-sample indicated stronger effects but also had much broader confidence intervals.

Data from the ACT study allowed us to illustrate how sensitivity analyses can be used to explore robustness of hazard ratio estimates and their standard errors to outcome misclassification. However, our analysis has some limitations. The method proposed here for estimating assessment level sensitivity and specificity based on sensitivity and specificity assessed at the end of all follow-up assumes that accuracy of the evaluation does not change over time, which may be unrealistic if, for instance, older subjects are more or less likely to be misclassified. This assumption can be relaxed but results will be strongly dependent on the proposed functional form of the relationship between time and accuracy. We have proposed a model using constant accuracy which would be appropriate in the absence of strong evidence supporting any particular alternative functional form for the relationship. We have also demonstrated an existing discrete-time approach which is appropriate for longitudinal studies under panel observation, but ignores variation in the timing of study visits and provides discrete-time hazard ratio estimates which correspond only approximately to the more familiar hazard ratios of the continuous time Cox proportional hazards model. Additionally, characteristics of patients who consent to autopsy and are deceased may differ from those of patients who are still alive [31]. This could affect estimates of sensitivity and specificity based on the autopsy sub-sample. We have therefore explored a range of values for sensitivity and specificity around the estimated values. Finally, we found that the likelihood was too flat to obtain maximum likelihood estimates for values of *ϕ* less than 0.97. This reflects the substantial uncertainty arising due to imperfect specificity. Sensitivity of results under outcome misclassification to imperfect specificity has been previously described [6]. Intuitively, for a rare event, even reasonably good specificity values can result in observed events consisting of more false-positives than true-positives, leading to severe uncertainty in estimated hazard ratios. Estimates of confidence interval inflation based on *ϕ* = 0.97 are thus likely to be underestimates of the true inflation factor.

## Conclusion

Outcome misclassification has the potential to substantially bias study results. The implications of misclassification for a given study can be explored through sensitivity analysis. Even in the absence of empirical information on sensitivity and specificity of the imperfect outcome, sensitivity analyses can be undertaken to quantify the robustness of results under a range of plausible values. Such analyses aid appropriate interpretation of study results and should be included as part of the standard analysis for imperfect time to event outcomes.
